# EmCyclinD-EmCDK4/6 complex is involved in the host EGF-mediated proliferation of *Echinococcus multilocularis* germinative cells *via* the EGFR-ERK pathway

**DOI:** 10.3389/fmicb.2022.968872

**Published:** 2022-08-04

**Authors:** Chonglv Feng, Zhe Cheng, Zhijian Xu, Ye Tian, Huimin Tian, Fan Liu, Damin Luo, Yanhai Wang

**Affiliations:** ^1^State Key Laboratory of Cellular Stress Biology, Faculty of Medicine and Life Sciences, School of Life Sciences, Xiamen University, Xiamen, Fujian, China; ^2^Parasitology Research Laboratory, School of Life Sciences, Xiamen University, Xiamen, Fujian, China; ^3^Medical College, Xiamen University, Xiamen, Fujian, China

**Keywords:** *Echinococcus multilocularis*, germinative cells, cyclin-dependent kinases, cyclin, proliferation

## Abstract

The larval stage of the tapeworm *Echinococcus multilocularis* causes alveolar echinococcosis (AE), one of the most lethal helminthic infections in humans. The tumor-like growth and development of the metacestode larvae within host organs are driven by a population of somatic stem cells, the germinative cells, which represent the only proliferative cells in the parasite. Host-derived factors have been shown to promote germinative cell proliferation. Since cells sense the external signal mainly in G1 phase of the cell cycle, host factors are expected to exert impacts on the machinery regulating G1/S phase of the germinative cells, which still remains largely unknown in *E. multilocularis*. In this study, we described the characterization of two key members of the G1/S phase cell-cycle regulation, EmCyclinD and EmCDK4/6. Our data show that EmCyclinD and EmCDK4/6 display significant sequence similarity to their respective mammalian homologs, and that EmCyclinD interacts with EmCDK4/6, forming a kinase-active complex to activate its substrate Rb1. EmCyclinD was actively expressed in the germinative cells. Addition of human EGF caused an elevated expression of EmCyclinD while inhibition of the EGFR-ERK signaling pathway in the parasite reduced the expression of EmCyclinD and downstream transcriptional factors. Treatment with Palbociclib, a specific CDK4/6 inhibitor, downregulated the expression of cell cycle-related factors and impeded germinative cell proliferation and vesicle formation from protoscoleces. Our data demonstrated that the EmCyclinD-EmCDK4/6 complex participates in the cell cycle regulation of germinative cells which is mediated by host EGF via the EGFR-ERK-EmCyclinD pathway in *E. multilocularis*.

## Introduction

Alveolar echinococcosis (AE) is the most lethal helminthic infection caused by *Echinococcus multilocularis*. AE poses a serious threat to human health (Eckert and Deplazes, 2004) and lacks effective methods of prevention as with other zoonotic diseases (Vazquez-Chagoyan et al., 2011; Tang et al., 2022; Yin et al., 2022). From a public health perspective, AE infection is usually caused by pathogens spillover events from domestic or wild animal species, especially exposed to the larval stage parasites (Dharmarajan et al., 2022). The larval stage of *E. multilocularis* grows as diffuse infiltration in the liver of patients, and eventually affects the entire liver, and may metastasizes to the brain, lungs and other important organs. The neoblasts of the free-living flatworm planarian are a population of pluripotent somatic stem cells with unlimited capacity for self-renewal and differentiation potential and are fundamental to planarian growth and development, as well as tissue renewal and regeneration (Pellettieri and Sanchez, 2007; Eisenhoffer et al., 2008; Rink, 2013). Cells with similar functions and renewal mechanisms have also been found in parasitic flatworms, the trematode and tapeworm (Koziol et al., 2010, 2014; Collins et al., 2013; Wang et al., 2013). In *E. multilocularis*, these cells are called germinative cells and are the only cells with proliferative capacity and differentiation potential in the larvae (Koziol et al., 2014). The germinative cells are the basis for the continuous growth and development of the alveolar hydatid in the host (Brehm, 2010a; Koziol et al., 2014).

Host factors are considered to interact with the corresponding receptors in *E. multilocularis* and activate the associated highly conserved signaling pathways, thereby affecting the growth and development of the parasite (Brehm and Spiliotis, 2008; Brehm, 2010b). Recent studies reported that host insulin and FGF (fibroblast growth factor) can stimulate the proliferation of germinative cells and the growth of metacestode vesicles (Hemer et al., 2014; Forster et al., 2019). We also demonstrated that host EGF can promote germinative cell proliferation and vesicle development by activating the EGFR/ERK signaling pathway in alveolar hydatid (Cheng et al., 2017). Since cell proliferation requires the accurate control of cell cycle, it is expected that the machinery implicated in regulation of the cell cycle of germinative cells is influenced by host-derived factors.

The cell cycle regulatory network, comprising the cyclin-dependent kinases (CDKs), Cyclin, and cyclin-dependent kinase inhibitors (CDIs), is a key mechanism determining the progression of cell proliferation, differentiation, senescence and death. This network precisely regulates the cell cycle by activating or inhibiting downstream signaling molecules of Cyclin-CDK complexes. In addition, negative regulation of CDIs are also essential for maintaining network precision. The control of the cell cycle initiation generally occurs in the G_1_ phase (Duronio and Xiong, 2013). When cells receive the mitogenic signals, they initiate the synthesis of Cyclin D, which in turn forms active complexes with CDK4 or CDK6. The complexes phosphorylate members of the retinoblastoma (Rb) protein family. Rb proteins inhibit the transcription of cell cycle-related factors by binding and modulating transcription factors such as E2F family members. One of the most crucial targets of Rb–E2F complexes is Cyclin E. Cyclin E-CDK2 complexes have been proposed to irreversibly inactivate Rb, so an event considered to be the passage of the cell to the restriction point (R-point) during G1 in which cells no longer require mitogenic stimuli to undergo cell division (Malumbres and Barbacid, 2005). It is assumed that the level of CyclinD determines the onset of cell cycle functioning (Lim and Kaldis, 2013). The protein families of Cyclin and CDK as well as related signaling pathways are conserved in evolution and have been shown to play a key role in regulating somatic stem cells in *Drosophila*, nematodes, and planarian (Fernandez-Taboada et al., 2010; Zhu and Pearson, 2013; Hetie et al., 2014; Ishidate et al., 2014).

In this study, we identified two key cell cycle regulators, EmCyclinD and EmCDK4/6 in *E. multilocularis*. Our data demonstrated that EmCyclinD forms an active complex with EmCDK4/6. Targeting the EGFR-ERK-EmCyclinD pathway can inhibits host EGF-stimulated germinative cell proliferation, suggesting EmCyclinD and EmCDK4/6 as druggable targets for the development of chemotherapeutics against AE.

## Materials and methods

### Ethics statement

All animal experiments were conducted in strict accordance with China regulations on the protection of experimental animals (Regulations for the Administration of Affairs Concerning Experimental Animals, version from 18 July, 2013) and specifically approved by the Institutional Animal Care and Use Committee of Xiamen University (Permit Number: 2013–0053).

### Identification and cloning of CyclinD and CDK4/6 gene of *Echinococcus multilocularis*

Published sequences of CDK4, CDK6, and CyclinD of human, mouse, *Drosophila*, zebrafish, *Xenopus*, and *C. elegans* (Supplementary Table 1) were used as queries to BLAST the *E. multilocularis* genome database (Tsai et al., 2013) available at Wormbase database.^1^ EmuJ_000456500 and EmuJ_001021000 were then identified as the homologs of CyclinD and CDK4/6, respectively, and their full coding sequences were amplified from the cDNA preparations as described previously (Brehm et al., 2000). Kinase domain and cyclin characteristic domain were determined using the online software SMART.^2^ The phylogenetic tree was generated by the neighbor-joining method (bootstrap = 1,000) using MEGA 7.0. Primers for amplification of the full coding sequences of EmCyclinD and EmCDK4/6 were used as shown in Supplementary Table 2.

### Parasite *in vitro* cultivation and drug treatment

*In vitro* cultivation and drug treatment of *E. multilocularis* metacestode vesicles were performed as previously described (Cheng et al., 2017). Briefly, for the growth assay, vesicles were cultured in the host cell conditioned medium supplemented with drug and the growth of vesicles was analyzed at indicated time. For the vesicle formation assay, protoscoleces were collected from parasite material and *in vitro* cultured in conditioned medium supplemented with drugs. The initial process of vesicle formation, in which protoscoleces dilate and vacuolate, were examined after 21 days of culture. The CDK4/6 inhibitor Palbociclib, EGFR inhibitor CI-1033, MEK inhibitor U0126 and hydroxyurea were supplied by Selleck Chemicals. Recombinant human EGF was supplied by PeproTech. Drugs were added into the culture medium at a final concentration as required. The vesicles were then used for RNA isolation, whole protein extraction, or EdU labeling at the indicated time.

### Co-immunoprecipitation and western blot

HEK-293T cell was conserved by the State Key Laboratory of Cellular Stress Biology, Xiamen University, China. EmCyclinD and EmCDK4/6, tagged at their C-terminus with Myc-tag or HA-tag, respectively, were sub-cloned into pcDNA3.3 plasmid (gifts from Prof. Han Jiahuai, Xiamen University, China). The expression plasmids were co-transfected into the HEK-293T cells with the aid of Turbofect Transfection Reagent (Thermo Scientific). Cell lysates were harvested at 36 h post-transfection using RIPA lysis buffer (Beyotime, China). Co-immunoprecipitation experiments were performed using anti-Myc or anti-HA antibodies conjugated Sepharose Beads (#3,400 and #3,956, Cell Signaling Technology). The lysates from experiments were subjected to 10% SDS-PAGE and transferred electrically to PVDF membranes. Detection of tagged proteins was performed using anti-Myc and anti-HA antibodies (#2,278 and #3,724, Cell Signaling Technology). The membranes then reacted in an enzyme-linked immunoassay with anti-rabbit IgG conjugated with HRP (Invitrogen) and subsequently detected using ECL according to the manufacturer’s instructions.

### *In vitro* kinase assay

*In vitro* kinase activity was determined by measuring the rate of ADP production in the *in vitro* kinase reaction using the ADP-Glo Kinase Assay Kit (Promega). Briefly, the EmCDK4/6-EmCyclinD complex was purified by Co-immunoprecipitation as the kinase (Fry et al., 2004) and then incubated with 3 μg of recombination human Rb1 protein (Proteintech) as the substrate in the experiment. The kinase reactions were performed with 1X Kinase Reaction Buffer A [40 mM Tris (pH 7.5), 20 mM MgCl_2_, 0.1 mg/mL BSA], 25 μM ATP, 1 mM DTT and incubated at RT, 20 min. The reactions were terminated by ADP-Glo reagent and then detected according to the manufacturer’s instructions. Each data point was collected in duplicate.

### mRNA expression analysis of EmCyclinD and downstream factors

Total RNA was extracted from metacestode vesicles treated with different drugs and then reverse transcribed into cDNA as previously described (Cheng et al., 2017). cDNAs were processed for real-time quantitative (qPCR) analysis using the primers as listed in Supplementary Table S2. The constitutively expressed gene *elp* was used as the internal control (Brehm et al., 2003).

### EmCyclinD polyclonal antibody preparation and western blot assay

The polyclonal antibody against EmCyclinD was prepared by immunizing New Zealand Rabbit with the synthetic peptide “CASAPNGSSNSRKHS” of EmCyclinD (Genscript, China). Purification of the EmCyclinD antibody from the antiserum was performed further by protein A and peptide affinity chromatography. Western blot was performed using the EmCyclinD antibody with a dilution of 1:200.

### Molecular docking

The EmCDK4/6 and Palbociclib docking analysis was performed by AutoDock (version 4.2). The Palbociclib three-dimensional structure was retrieved by the Pubchem website^3^ and the EmCDK4/6 three-dimensional structure was built with online tool Swiss-model.^4^ The AutoDock software was adopted for EmCDK4/6 protein hydrogenation processing, calculating charge, and setting the receptor proteins docking lattice parameter. We eventually obtained 10 conformations about the interaction between EmCDK4/6 and Palbociclib. The three-dimensional diagram of the interaction between EmCDK4/6 and Palbociclib was displayed by PyMOL (version 2.4.0a0).

### 5-ethynyl-2′-deoxyuridine labeling

*In vitro* cultivated metacestode vesicles were treated with different drugs as required and then incubated with 50 μM of EdU for 4 h and whole-mount prepared as described before (Cheng et al., 2015). The detection of EdU was using Click-iT EdU Alexa Fluor 555 or Alexa Fluor 488 Imaging KIT (Life Technologies). DNA was counterstained with 4′,6-diamidino-2-phenylindole (DAPI) after EdU detection. For the quantification of EdU^+^ cells, at least four random microscopic fields from at least five vesicles were captured and the positive cells were counted manually. The labeling experiments were repeated at least two times and analyzed for each control and treatment group.

### Data analyses and statistics

Data was shown as mean ± *SD* as indicated in the respective figure legend. Data from the control and experimental groups were compared, and the significance was determined using two-tailed Student’s *t*-test.

## Results

### Identification of EmCyclinD and EmCDK4/6

**FIGURE 1:**
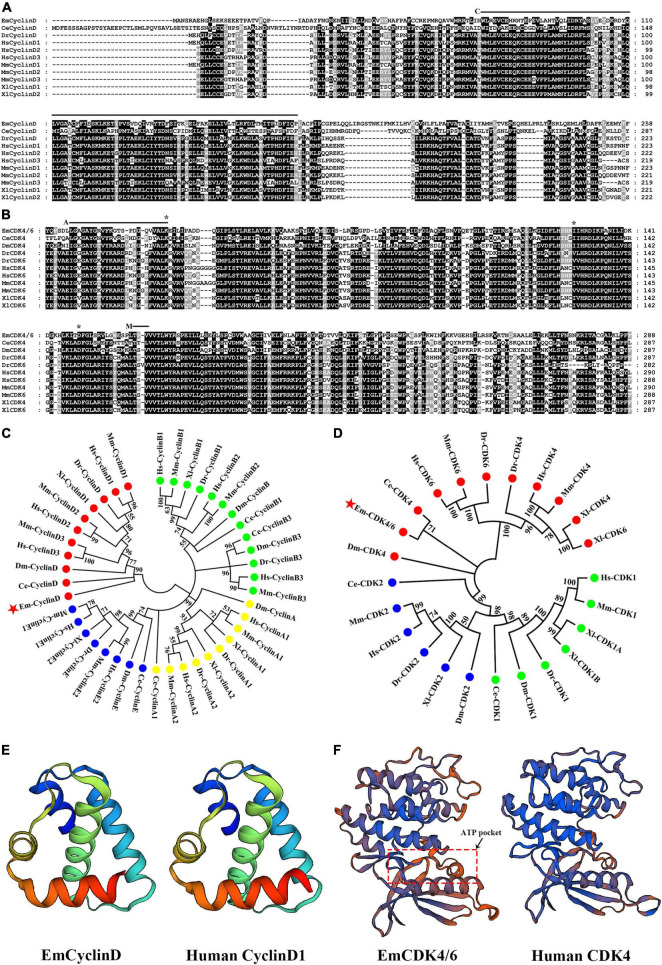
Identification of CyclinD and CDK4/6 homologs in *E. multilocularis*. **(A)** Alignment of EmCyclinD with other established CyclinD members. The Cyclin box domain is marked with “C.” **(B)** Alignment of the S_Tkc domain of EmCDK4/6 with that of other CDK4 or 6 members. The ATP binding site is marked with “A,” the phosphorylation motif is marked with “M” and predicted Palbociclib binding sites are marked with “*.” **(C,D)** Phylogenetic analysis of EmCyclinD and EmCDK4/6 (marked with red stars). The full-length sequence of CyclinD members and the S_Tkc domain of CDK4/6 members were used for phylogenetic tree construction. Em, *Echinococcus multilocularis*; Ce, *Caenorhabditis elegans*; Hs, *Homo sapiens*; Mm, *Mus musculus*; Dm, *Drosophila melanogaster*; Dr, *Danio rerio*; Xl, *Xenopus laevis*. **(E)** Comparison of the predicted three-dimensional structures of the Cyclin box of human CyclinD (UniprotKB code P24385) and EmCyclinD. **(F)** Comparison of the predicted three-dimensional structures of the S_Tkc domain of human CDK4 (UniprotKB code P11802) and EmCDK4/6.

We excavated the genome information of *E. multilocularis* by BLAST analyses using human and mouse CyclinD1, D2, D3, or CDK4, 6 as the queries and procured as best hit proteins encoded by locus EmuJ_000456500 (annotated as G1: S specific cyclin D1) and EmuJ_001021000 (annotated as Cyclin-dependent kinase 6). Phylogenetic analysis revealed that EmuJ_000456500 could be classified as *D*-type cyclin group (Figures 1A,C) and EmuJ_001021000 is closely related to the CDK4 and CDK6 group (Figures 1B,D). Then EmuJ_000456500 was named *EmCyclinD* and EmuJ_001021000 was named *EmCDK4/6.*

The inferential EmCyclinD protein is comprised of 386 amino acids with a predicted molecular weight of 43.2 kDa. *EmCyclinD* locus consists of 4 exons and 3 introns distributed in the genome region of 1,334 bps with a coding sequence length of 1,161 bps. A conserved cyclin characteristic domain Cyc_N (or cyclin box) which contains the CDK-binding site is located between residues 72 and 156 (marked “C” in Figure 1A) (Nugent et al., 1991). The Cyc-C domain, which is typical for mammalian CyclinD, is absent in *E. multilocularis*, similar to the *C. elegans* ortholog (Kitagawa et al., 1996).

*EmCDK4/6* which occupies the genome region of 2,884 bps is made up of 6 exons and 5 introns. The coding sequence comprised 2,136 bps. *EmCDK4/6* has over 1,000 bps additional C-terminal extension compared to *CDK4* or *CDK6* of human or other model species. To investigate possible alternative splicing events, we performed 3’ RACE on cDNA from metacestode vesicles but detected only one single transcript. The EmCDK4/6 protein contains 711 amino acids with a calculated molecular weight of 77.0 kDa. Structural analysis showed that EmCDK4/6 possesses the conserved CDK domains and functional amino acid sites of CDK4 and CDK6 proteins, including the Serine/Threonine kinase catalytic domain (S_TKc, positions 24–311, Figure 1B) and the phosphorylation motif Ser/Thr-Pro-X (positions 187–189, marked “M” in Figure 1B), so it is equibed with the typical features of CDK4 or 6 proteins (Matsushime et al., 1992; Kitagawa et al., 1996; Park and Krause, 1999).

The analysis of three-dimensional structure revealed that EmCyclinD Cyc_N domain has similar α-helix structures as human CyclinD (Figure 1E). EmCDK4/6 has a bilobed flap shape in S_Tkc domain which contains the amino end (bottom of the model) rich in β-folds and the carboxyl end (top of the model) rich in α-helices. The ATP binding site is located in the cleft between the domains, showing a pocket-like structure (Figure 1F; Day et al., 2009).

### EmCDK4/6 interacts with EmCyclinD and activates Rb1 *In vitro*

**FIGURE 2:**
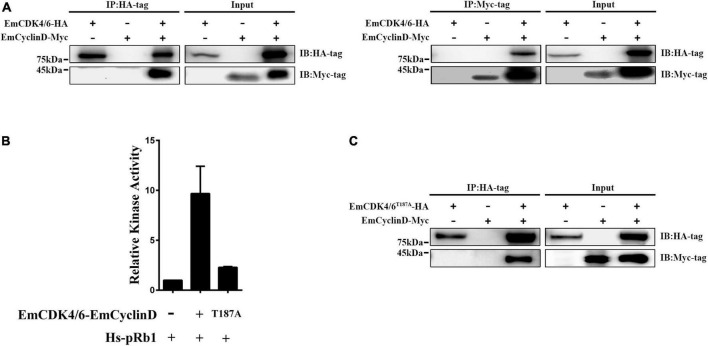
EmCDK4/6 interacts with EmCyclinD and displays *in vitro* kinase activity. **(A)** Co-Immunoprecipitation of HA-tagged EmCDK4/6 with Myc-tagged EmCyclinD. The left shows the results using the anti-HA tag antibody for immunoprecipitation and the right using the anti-Myc tag antibody. **(B)** *In vitro* kinase assay of the EmCyclinD-EmCDK4/6 complex or EmCyclinD-EmCDK4/6^T187A^ complex. Data are shown as mean ± *SD* of duplicate. **(C)** Co-Immunoprecipitation of EmCyclinD with EmCDK4/6^T187A^ mutant. Note that the mutation T187A does not affect the binding of EmCyclinD to EmCDK4/6.

In mammals, cyclin D generally binds to CDK4 or 6 during G1/S phase and then the CyclinD-CDK4/6 complex phosphorylates downstream Rb1 proteins to release transcriptional factors such as E2F (Sherr and Roberts, 1999; Choi and Anders, 2014). Our data revealed that EmCyclinD co-immunoprecipitates with EmCDK4/6 *in vitro* (Figure 2A), suggesting an interaction between EmCyclinD and EmCDK4/6. By mining the genome database, we found that *E. multilocularis* also possesses a CDK2 homolog (EmuJ_000258800, referred to as *EmCDK2* in this study) and that EmCyclinD could not interact with EmCDK2 (Supplementary Figure 1). These results suggest that EmCyclinD may specifically form a complex with EmCDK4/6 but not with other CDKs in *E. multilocularis*, in line with the previous findings in other species (Wianny et al., 1998).

To further investigate whether the EmCDK4/6-EmCyclinD complex possesses enzymatic activities, we performed *in vitro* kinase assays using human Rb1 as the substrate. As shown in Figure 2B, the EmCDK4/6-EmCyclinD complex displayed kinase activity against human Rb1. In mammalian cells, the activity of CDK4/6 depends on the Ser/Thr-Pro-X motif within the Serine/Threonine kinase catalytic domain, whereas mutations of the Ser/Thr residue in this motif do not affect the binding of CDK4/6 to CyclinD (Kato et al., 1994). We then generated an inactive form of EmCDK4/6 by substituting alanine for threonine (T187A). The results show that EmCDK4/6^T187A^ could complex with EmCyclinD but the kinase activity was greatly reduced (Figures 2B,C), suggesting the necessity of Thr187 for EmCDK4/6’s kinase function. Together, these results demonstrated that EmCDK4/6 interacts with EmCyclinD and possesses enzymatic activities.

### Expression of EmCyclinD in *Echinococcus multilocularis* larvae

**FIGURE 3:**
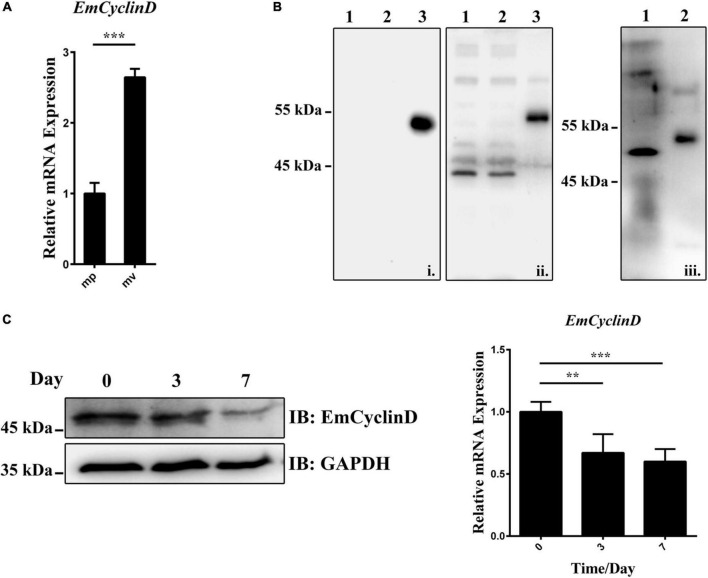
Expression of EmCyclinD in *E. multilocularis* germinative cells. **(A)** Relative mRNA levels of EmCyclinD in the mature protoscoleces (mp) and metacestode vesicles (mv). **(B)** Lysates of bacteria expressing His-tagged EmCyclinD were analyzed by western blotting using the anti-His tag antibody (i) or the anti-EmCyclinD antibody (ii). Line 1 and 4: empty vector control; line 2 and 5: IPTG uninduced; line 3 and 6: IPTG induced. Total protein of *in vitro*-cultured vesicles (iii, line 7) was analyzed with the anti-EmCyclinD antibody. His-tagged recombinant EmCyclinD was used as a control (iii, line 8). **(C)** mRNA and protein expressions of EmCyclinD in the metacestode vesicles treated with 40 mM of hydroxyurea for 3 or 7 days. Data in **(A,C)** are shown as mean ± *SD*. **P < 0.01, ***P < 0.001.

Given that the germinative cells are the only proliferative cells in *E. multilocularis*, we speculated that EmCyclinD would be highly expressed in the actively proliferating germinative cells. We then analyzed EmCyclinD expression in metacestode vesicles which have a large population of proliferating germinative cells and in the mature protoscoleces in which the germinative cells maintain slow cell cycle kinetics or remain in a quiescent state (Koziol et al., 2014). As shown in Figure 3A, we found that the mRNA level of *EmCyclinD* was much higher in the metacestode vesicles than in the mature protoscoleces (Figure 3A).

We also generated a polyclonal antibody against EmCyclinD, which effectively detected the recombinant His-tagged EmCyclinD protein as well as the endogenous EmCyclinD in the *in vitro*-cultivated vesicles (Figure 3B). We then explored the protein expression of EmCyclinD in the vesicles treated with hydroxyurea (HU). HU is a DNA synthesis inhibitor and has been shown to arrest the cell cycle at G1/S phase (Timson, 1975) and specifically to deplete the germinative cell populations in *E. multilocularis* (Koziol et al., 2014). As shown in Figure 3C, we found that both of the mRNA and protein levels of EmCyclinD were lower after HU treatment. Taken together, these results suggest that EmCyclinD is actively expressed in the proliferating germinative cells of *E. multilocularis.*

### EmCyclinD-EmCDK4/6 complex participates in the *Echinococcus multilocularis* germinative cell proliferation *via* EGFR-ERK pathway

**FIGURE 4:**
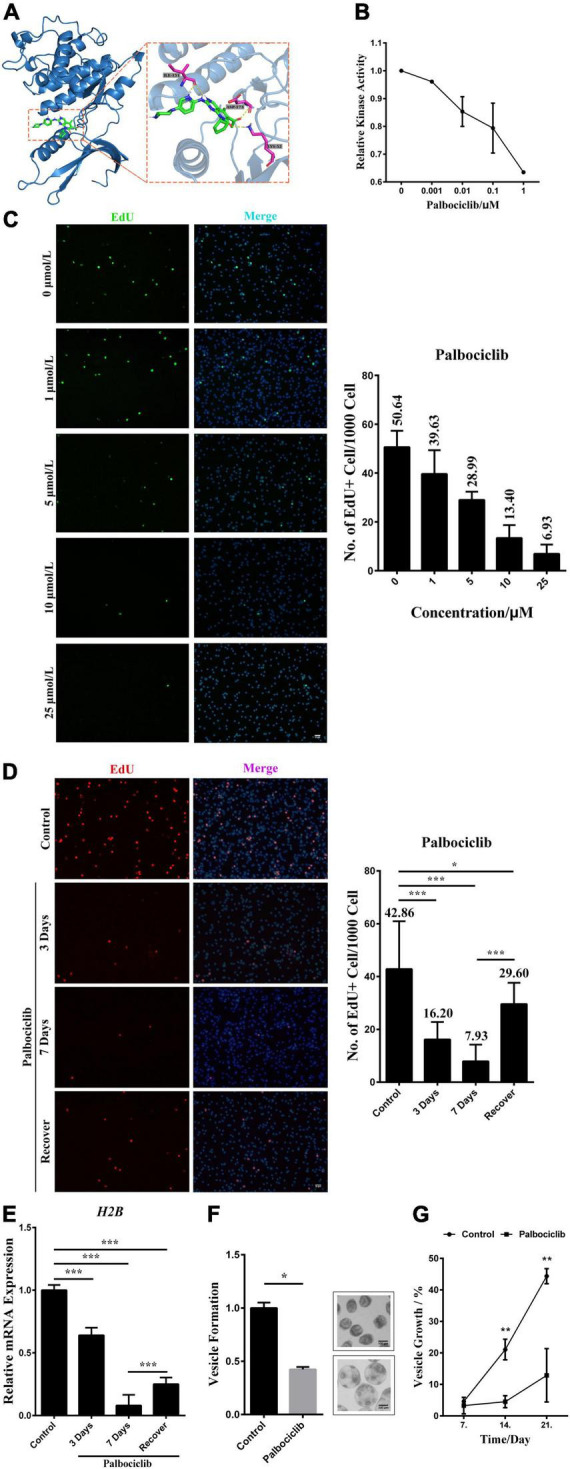
EmCyclinD-EmCDK4/6 complex participates in regulating germinative cell proliferation in *E. multilocularis.* **(A)** Docking structure and interactions between EmCDK4/6 and Palbociclib. The left shows that Palbociclib binds to the ATP binding pocket of EmCDK4/6. The right shows the details of the EmCDK4/6-Palbociclib interaction. **(B)** *In vitro* kinase assay of EmCyclinD-EmCDK4/6 complex with Palbociclib. Data are shown as mean ± *SD* of duplicate. **(C)** 3 days effects of different concentrations of Palbociclib on germinative cell proliferation. Representative images are shown in the left panel, Bar = 50 μm. Quantification of EdU + cells is shown in the right panel. Data are shown as mean ± *SD* of 2 separate labeling experiments. EdU detection was performed using green fluorophore (Alexa Fluor 488). **(D)** Proliferation recovery after 7 days of 10 μM Palbociclib treatment. Metacestode vesicles were allowed for recovery in the absence of drugs for 1 day. Representative images are shown in the left panel, Bar = 50 μm. Quantification of EdU + cells is shown in the right panel. *P < 0.05, ***P < 0.001. Data are shown as mean ± *SD* of 12 vesicles. EdU detection was performed using red fluorophore (Alexa Fluor 555). **(E)** mRNA expression of downstream H2B factor upon Palbociclib treatment. Data are shown as mean ± *SD*. ***P < 0.001. **(F)** 10 μM Palbociclib inhibits the process of vesicle formation from protoscoleces after 21 days treatment. Control was set to 1 and results were normalized against the control. Representative images of vesicle formation are shown on the right. Upper: protoscoleces collected from parasite material. Lower: protoscoleces undergoing vesicle formation **(G)** 10 μM Palbociclib inhibits the growth of metacestode vesicles. Metacestode vesicles growth is shown as the increase of vesicle diameter as compared to day 0. Comparison between the Palbociclib treatment and the control at the same timepoint was performed using two-tailed Student’s *t*-test. **P < 0.01.

It has been shown that Palbociclib, a highly selective inhibitor of human CDK4/6, can bind to the ATP-binding pocket of CDK4/6. Consequently, it induces G1 arrest and a concomitant reduction of Rb1 phosphorylation (Fry et al., 2004; Chen et al., 2016). Considering the similarities between human CDK4 and EmCDK4/6 three-dimensional structures, we inferred that Palbociclib might also inhibit the activities of EmCDK4/6. The result of docking predictions demonstrated that Palbociclib exhibits the potential ability to bind to EmCDK4/6 (Figure 4A). Palbociclib integrated into the ATP binding pocket of EmCDK4/6 and involves 4 hydrogen bonds in 3 amino acids (i.e., Lys52, Ile151, and Asp173.), which are all conserved in human CDK6 (corresponding to Lys43, Val101 and Asp163, respectively, Figure 1B). These three amino acids are involved in the binding of Palbociclib to human CDK6 and the last two among them are considered to contribute mostly to the binding energy (Hernandez et al., 2017). The *in vitro* kinase assay also revealed that Palbociclib attenuated the kinase activity of EmCyclinD-EmCDK4/6 complex (Figure 4B).

We then treated metacestode vesicles with Palbociclib and labeled the proliferating germinative cells with EdU, an analog of thymidine. The results show that Palbociclib greatly reduced the number of EdU^+^ cells in a dose-dependent manner (Figure 4C). The number of proliferating germinative cells was decreased by ∼80% after 7 days of treatment with 10 μM of Palbociclib. With the consideration of Palbociclib as a reversible CDK inhibitor, we removed the drug after 7 days of treatment and found that the proliferation of germinative cells was mostly restored (Figure 4D). Furthermore, Palbociclib treatment inhibited the mRNA expression of *EmH2B*, a S-phase marker of the proliferating germinative cells (Figure 4E). These results suggest that Palbociclib may cause cell cycle arrest of the germinative cells. *Echinococcus* protoscoleces have a unique feature that they can mature into adult tapeworms in the definitive host (canids), or can undergo reverse development into metacestode vesicles if distributed in the intermediate host (Eckert et al., 1983; Mehlhorn et al., 1983; Koziol et al., 2014). When cultured in the conditioned medium, protoscoleces would tend to develop into vesicles (Hemer et al., 2014; Cheng et al., 2017). Our data show that the initial process of vesicle formation from protoscoleces and the growth of vesicles were also greatly inhibited by Palbociclib (Figures 4F,G). Taken together, these data suggest that Palbociclib inhibits the activity of the EmCyclinD-EmCDK4/6 complex, leading to impeded germinative cell proliferation and parasite growth.

**FIGURE 5:**
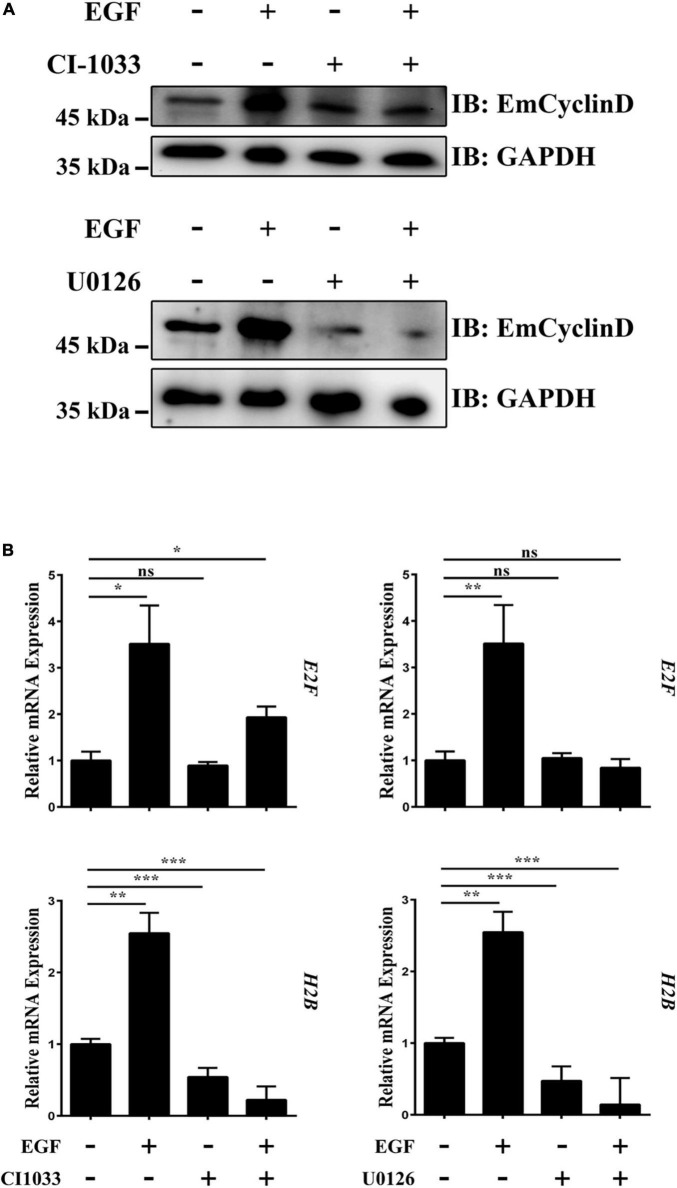
The EGFR-ERK pathway regulates EmCyclinD and downstream factors expression in *E. multilocularis.* **(A)** CI-1033 and U0126 inhibit EGF-stimulated EmCyclinD expression in *E. multilocularis*. Metacestode vesicles were treated with 10 μM CI-1033 or 40 μM U0126 for 3 h and then stimulated with EGF for 45 min. **(B)** mRNA expressions of EmCyclinD and S-phase related factors after CI-1033 or U0126 treatment. Data are shown as mean ± *SD*. *P < 0.05, **P < 0.01, ***P < 0.001. ns, *P* > 0.05.

The expression and accumulation of Cyclin D are induced by growth factors such as EGF (Musgrove, 2006). In this study, we found that EmCyclinD expression was up-regulated upon host EGF stimulation (Figure 5A and Supplementary Figure 2), suggesting that host EGF affects the cell cycle of germinative cells. It has been shown that the EGFR/ERK signaling pathway in *E. multilocularis* responds to host EGF and promotes germinative cell proliferation (Cheng et al., 2017). We then treated vesicles with the EGFR inhibitor CI-1033 or the MEK/ERK inhibitor U0126 in the presence or absence of EGF. The results showed that CI-1033 and U0126 not only reduced the basal levels of EmCyclinD in the vesicles but also greatly inhibited the EGF-induced upregulation of EmCyclinD (Figure 5A). E2F and Histone H2B (H2B) are known as the CyclinD downstream and S phase-related factor in mammalian cells (Whitfield et al., 2006). Orthologs to these factors are also present in the *E. multilocularis* genome: E2F (EmuJ_000535700, annotated as transcription factor E2F4), H2B (EmuJ_000387800, annotated as histone H2B). We found that, along with the downregulation of EmCyclinD, inhibition of EGFR and MEK also decreased the transcriptional levels of E2F and H2B (Figure 5B). Meanwhile, the proliferation of germinative cells in the vesicles was promoted upon EGF stimulation while inhibited upon the treatment with CI-1033 or U0126 (Supplementary Figure 3), consistent with our earlier research. Taken together, these results suggest that EmCyclinD and downstream cell cycle-related factors expression levels could be regulated by the host EGF-mediated EGFR-ERK signaling pathway.

## Discussion

Cyclin-CDK complex play a key role in orchestrating the complex events of cell cycle. In the matter of the G1 phase, as long as the cells sense the mitogenic signals from external cues, Cyclin D accumulates, associates with and activates CDK4/6 to initiate the cell cycle. This mechanism of cell-cycle regulation has been extensively documented in various free-living animals and is considered conserved across metazoans; however, the situation is somewhat different for the metacestode larvae of *E. multilocularis*. In the light of the intimate contact with host tissues and the long-term co-evolutionary process, the parasite is believed, and indeed proved by multiple lines of evidence, capable of sensing the host-derived signals that in turn exert an influence on the germinative cells (Vicogne et al., 2004; Hemer et al., 2014; Cheng et al., 2017; Forster et al., 2019). Since the germinative cells are the only cells that undergo cell division cycle in *E. multilocularis*, this information therefore provides clues regarding the possibility that host factors have been implicated in regulating Cyclin-CDK complex and cell cycle in the parasite.

In this study, we describe two important molecules, EmCyclinD and EmCDK4/6, which are involved in the G1/S-phase regulation in *E. multilocularis*. In comparison with their mammalian homologs, EmCyclinD and EmCDK4/6 are highly conserved in terms of functionally relevant domains (Figure 1). While EmCyclinD lacks the CYC_C domain like *C. elegans* CyclinD (Kitagawa et al., 1996). Moreover, EmCDK4/6 has a functional unknown sequence at its carboxyl terminus which was predicted to be randomly coiled. Similar sequences have not been identified in other species and the role remains to be investigated.

Our data show that EmCyclinD is actively expressed in the proliferating germinative cells and could interact with EmCDK4/6 to form a complex which displayed the kinase activity of phosphorylating human Rb1 protein *in vitro* (Figures 2, 3). During the G1 phase of mammalian cells, Rb1 is the most crucial downstream effector of the CyclinD-CDK4/6 complexes, phosphorylated by the complexes at Ser780, Ser795, Ser807, and Ser811, and then allows progression of the cell cycle from G1 to S phase (Rubin, 2013). *E. multilocularis* possesses a single Rb1 homolog (encoded by EmuJ_000159800, referred to as EmRb1 in this study) and 3 of the phosphorylation sites (T910, T922 and T926, corresponding to S795, S807 and S811 of Human Rb1, respectively) related to CyclinD-CDK4/6 were well conserved (Supplementary Figure 4). We could also identify a single E2F member in *E. multilocularis* (encoded by EmuJ_000535700, referred to as EmE2F), indicating that the conserved CyclinD-CDK4/6-Rb1-E2F signaling cascade may also exist in the parasite. Interestingly, we found that EmCyclinD could not complex with EmCDK2, suggesting a specific interaction between the Cyclins and their corresponding CDKs in *E. multilocularis* (Supplementary Figure 1). An investigation identifying the Cyclin that associates with EmCDK2 is currently being carried out in our laboratory.

We have previously defined that the EGFR/ERK signaling pathway in *E. multilocularis* responds to host EGF and promotes germinative cell proliferation (Cheng et al., 2017). We herein deepen our understanding of this mechanism. Despite failing to detect the changes in the phosphorylation of the downstream effector EmRb1 due to unavailable antibodies, we show that the transcriptional levels of the S-phase related factor were elevated upon host EGF stimulation, accompanied by the promoted EmCyclinD expression and germinative cell proliferation (Figure 5 and Supplementary Figure 3). Since EmCyclinD could form a complex with EmCDK4/6 and inhibition of either the complex or the EGFR/ERK signaling resulted in attenuated effects of EGF on EmCyclinD expression (Figures 4, 5), our data suggest that host EGF is implicated in regulating the activity of EmCyclinD-EmCDK4/6 complex through the EGFR/ERK signaling, which in turn influences the cell cycle progression and the proliferation of germinative cells. Although these findings are mainly based on *in vitro* experiments, our data also demonstrated that the physiologically relevant concentrations of EGF (1–10 ng/mL) could apparently induce the expression of EmCyclinD in the cultivated metacestode vesicles (Supplementary Figure 2), and therefore it is conceivable that a similar situation may occur in the growing and developing parasite within the host.

Increasing evidence has shown that a number of kinase inhibitors originally designed for the treatment of human tumors also display effects on parasitic helminths including *E. multilocularis*, owing to the structural similarities between the parasite’s kinases and their mammalian homologs (Graeser et al., 1996; Santori et al., 2002; Augusto et al., 2018). Palbociclib has been proved as a potent inhibitor of human CDK4/6 and few reports have demonstrated its activity against other kinases. Generally, 0.1–1 μM of Palbociclib reduces Rb1 phosphorylation, inhibits cell proliferation and even causes cell death in a variety of cell lines (Menu et al., 2008; Finn et al., 2009; Katsumi et al., 2011; Kwong et al., 2012; Salvador-Barbero et al., 2020). While there are a little research concerning the effects of Palbociclib on non-mammalian animals, the considerable concentration for the CDK4/6-inhibition phenotype in zebrafish is 50 μM (Liu et al., 2011). In the present study, we showed that Palbociclib inhibited the activity of EmCyclinD-EmCDK4/6 complex *in vitro* and 5 μM or higher concentrations of drugs significantly inhibited the proliferation of germinative cells (Figure 4). It was reported that Palbociclib, in spite of being considered a reversible CDK inhibitor, causes cytotoxic effects and tumor regression with long-term treatment (Leonard et al., 2012; Dickson et al., 2013; Altenburg and Farag, 2015; Goel et al., 2016). Our data show that long-term treatment of Palbociclib could effectively reduce the metacestode vesicle formation and growth (Figure 4), suggesting EmCDK4/6 as a druggable target for new drug development against AE.

In conclusion, we described the characterization of EmCyclinD and EmCDK4/6 and showed that host EGF activates EmCyclinD-EmCDK4/6 complex via the EGFR/ERK signaling in *E. multilocularis*, which in turn regulates germinative cell proliferation and parasite growth. These findings will deepen our understanding of the stem cell biology of *E. multilocularis* and the complex host-parasite interaction, and it will be helpful for the development of novel chemotherapeutics against AE.

## Data availability statement

The datasets presented in this study can be found in online repositories. The names of the repository/repositories and accession number(s) can be found below: https://www.uniprot.org/uniprotkb/A0A068Y4X2/entry, EmCDK4/6 (EmuJ_000456500): A0A068Y4X2 and https:// www.uniprot.org/uniprotkb/A0A068YJR9/entry, EmCyclinD (EmuJ_001021000): A0A068YJR9.

## Ethics statement

The animal study was reviewed and approved by the Institutional Animal Care and Use Committee of Xiamen University.

## Author contributions

CF, ZC, and YW conceived and designed the experiments. CF and ZX performed the experiments. CF and ZC analyzed the data and wrote the manuscript. YT, HT, and FL offered writing proposals. All authors contributed to the article and approved the submitted version.

## Footnotes

1
https://parasite.wormbase.org/Echinococcus_multilocularis_prjeb122/Info/Index/

2
http://smart.embl-heidelberg.de/

3
https://pubchem.ncbi.nlm.nih.gov

4
https://swissmodel.expasy.org

## Supplementary material

Supplementary Figure 1 Analysis of the CDK2 homolog of *E. multilocularis* amino acid sequence and the interaction with EmCyclinD. **(A)** Alignment of the S_Tkc domain of EmCDK2 with multiple species CDK2. **(B)** Phylogenetic analysis of EmCDK2 (marked with red star). The BLAST analyses of the S_Tkc domain of EmCDK2 were used for phylogenetic tree construction. **(C)** The Co-Immunoprecipitation of EmCyclinD and EmCDK2. Using an Anti-HA tag antibody for Immunoprecipitation.

Supplementary Figure 2 Supplementary Figure 2 Effects of EGF on EmCyclinD expression. Metacestode vesicles were cultured in DMEM without serum for 5 days, and then stimulated with 0 (Control) to 100 ng/mL recombinant human EGF for 45 min.

Supplementary Figure 3 The inhibition of EGFR-ERK inhibits the proliferation of germinative cells. **(A)** Host EGF stimulates the proliferation of germinative cells. Metacestode vesicles were pretreated with 40 mM of hydroxyurea for 3 days and allowed for a 4 days’ recovery in conditioned medium (control) supplemented with EGF. **(B)** CI-1033 and U0126 treatment inhibit germinative cell proliferation. Metacestode vesicles were treated with 10 μM CI-1033 or 40 μM U0126 for 4 days, Representative images are shown in the left panel, Bar = 50 μm. Quantification of EdU + cells is shown in the right panel. **P* < 0.05, ^**^*P* < 0.01. Data are shown as mean ± *SD* of at least three vesicles.

Supplementary Figure 4 Analysis of amino acid sequence of EmRb1. Alignment of the full length of EmRb1 with Human Rb1. Three CyclinD-CDK4/6 complex-related phosphorylation sites are conserved (Orange box).
