# Supplementary material for: EmCyclinD-EmCDK4/6 complex is involved in the host EGF-mediated proliferation of Echinococcus multilocularis germinative cells via the EGFR-ERK pathway
Source: Front Microbiol. 2022 Aug 4;13:968872. doi: 10.3389/fmicb.2022.968872 (PMC9410764; doi:10.3389/fmicb.2022.968872)
Supplement: Supplementary file 1 [file Table_1.DOCX]

**Table.S1. The accession numbers of CyclinD, CDK4 and CDK6 for phylogenetic tree construction.**

| **Species** | **Protein Name** | **Accession Number (UniProt)** |
| --- | --- | --- |
| *Echinococcus multilocularis* | Em-CyclinD | A0A068Y4X2 |
|  | Em-CDK4/6 | A0A068YJR9 |
| *Caenorhabditis elegans* | Ce-CyclinD | Q9U2M5 |
|  | Ce-CyclinE | O01501 |
|  | Ce-CyclinA1 | P34638 |
|  | Ce-CyclinB1 | Q10653 |
|  | Ce-CyclinB3 | Q10654 |
|  | Ce-CDK4 | Q9XTR1 |
|  | Ce-CDK2 | O61847 |
|  | Ce-CDK1 | P34556 |
| *Homo sapiens* | Hs-CyclinD1 | P24385 |
|  | Hs-CyclinD2 | P30279 |
|  | Hs-CyclinD3 | P30281 |
|  | Hs-CyclinE1 | P24864 |
|  | Hs-CyclinE2 | O96020 |
|  | Hs-CyclinA1 | P78396 |
|  | Hs-CyclinA2 | P20248 |
|  | Hs-CyclinB1 | P14635 |
|  | Hs-CyclinB2 | O95067 |
|  | Hs-CyclinB3 | Q8WWL7 |
|  | Hs-CDK4 | P11802 |
|  | Hs-CDK6 | Q00534 |
|  | Hs-CDK2 | P24941 |
|  | Hs-CDK1 | P06493 |
| *Mus musculus* | Mm-CyclinD1 | P25322 |
|  | Mm-CyclinD2 | P30280 |
|  | Mm-CyclinD3 | P30282 |
|  | Mm-CyclinE1 | Q61457 |
|  | Mm-CyclinE2 | Q9Z238 |
|  | Mm-CyclinA1 | Q61456 |
|  | Mm-CyclinA2 | P51943 |
|  | Mm-CyclinB1 | P24860 |
|  | Mm-CyclinB2 | P30276 |
|  | Mm-CyclinB3 | Q810T2 |
|  | Mm-CDK4 | P30285 |
|  | Mm-CDK6 | Q64261 |
|  | Mm-CDK2 | P97377 |
|  | Mm-CDK1 | P11440 |
| *Drosophila melanogaster* | Dm-CyclinD | Q7KUZ5 |
|  | Dm-CyclinE | P54733 |
|  | Dm-CyclinA | P14785 |
|  | Dm-CyclinB | P20439 |
|  | Dm-CyclinB3 | Q9I7I0 |
|  | Dm-CDK4 | Q94877 |
|  | Dm-CDK2 | P23573 |
|  | Dm-CDK1 | P23572 |
| *Danio rerio* | Dr-CyclinD | Q90459 |
|  | Dr-CyclinE | P47794 |
|  | Dr-CyclinA1 | F1QGH4 |
|  | Dr-CyclinA2 | Q98TA3 |
|  | Dr-CyclinB1 | Q9IB44 |
|  | Dr-CyclinB3 | F1QRE5 |
|  | Dr-CDK4 | F1RDQ7 |
|  | Dr-CDK6 | E9QFH2 |
|  | Dr-CDK2 | Q7ZWB1 |
|  | Dr-CDK1 | Q7T3L7 |
| *Xenopus laevis* | Xl-CyclinD1 | P50755 |
|  | Xl-CyclinE2 | Q91780 |
|  | Xl-CyclinA1 | P18606 |
|  | Xl-CyclinB1 | P13350 |
|  | Xl-CDK4 | Q91727 |
|  | Xl-CDK6 | Q5HZQ2 |
|  | Xl-CDK2 | P23437 |
|  | Xl-CDK1A | P35567 |
|  | Xl-CDK1B | P24033 |
