# Supplementary material for: EmCyclinD-EmCDK4/6 complex is involved in the host EGF-mediated proliferation of Echinococcus multilocularis germinative cells via the EGFR-ERK pathway
Source: Front Microbiol. 2022 Aug 4;13:968872. doi: 10.3389/fmicb.2022.968872 (PMC9410764; doi:10.3389/fmicb.2022.968872)
Supplement: Supplementary file 2 [file Table_2.DOCX]

**Table.S2. The sequence of primers used in this study.**

| **Purposes** | **Primer Name** | **Sequence** |
| --- | --- | --- |
| Identification of *EmCDK4/6* and *EmCyclinD* | EmCDK4/6-F | 5’-ATG GCT AGC TTG AGG CCT TTA CT-3’ |
|  | EmCDK4/6-R | 5’-TTA GTT ATC ACT TGT TGA CGT CTG GT-3’ |
|  | EmCyclinD-F | 5’-ATG GCG AAC TCA AGG GCT GAA CA-3’ |
|  | EmCyclinD-R | 5’-TTA TGA GTG CTT CCT GGA GTT GGA GC-3’ |
| mRNA expression analysis | EmCyclinD-qF | 5’-GGA ATT GTT TGC AAA AGA GC-3’ |
|  | EmCyclinD-qR | 5’-GAT CCT CGA ATA AGC TGC TG-3’ |
|  | EmE2F-qF | 5’-GCA TTC AAT GGA AGG GTG GGA-3’ |
|  | EmE2F-qR | 5’-GGG GGC GCG AAT TAC TAC TAG A-3 |
|  | EmH2B-qF | 5’- CGA AGG CCG TGA CCA A-3’ |
|  | EmH2B-qR | 5’- CAG CCA CAA CAA CCA AGA AT-3’ |
|  | EmMCM2-qF | 5’-ACC GTA AAT GAG TGG G-3’ |
|  | EmMCM2-qR | 5’-TCG GGA AGG AAG TAA G-3’ |
|  | EmMCM6-qF | 5’-AAC GCA AAG GAA CGA G-3’ |
|  | EmMCM6-qR | 5’-CGG AAA GAG GGA GGT AT-3’ |
|  | elp-qF | 5’-CAG GAT CTC TTC GAT CAA GTG-3’ |
|  | elp-qR | 5’-GAC CAT ACT TGG CAA CAC AGG-3’ |
