# Supplementary figures and images for: EmCyclinD-EmCDK4/6 complex is involved in the host EGF-mediated proliferation of Echinococcus multilocularis germinative cells via the EGFR-ERK pathway
Source: Front Microbiol. 2022 Aug 4;13:968872. doi: 10.3389/fmicb.2022.968872 (PMC9410764; doi:10.3389/fmicb.2022.968872)

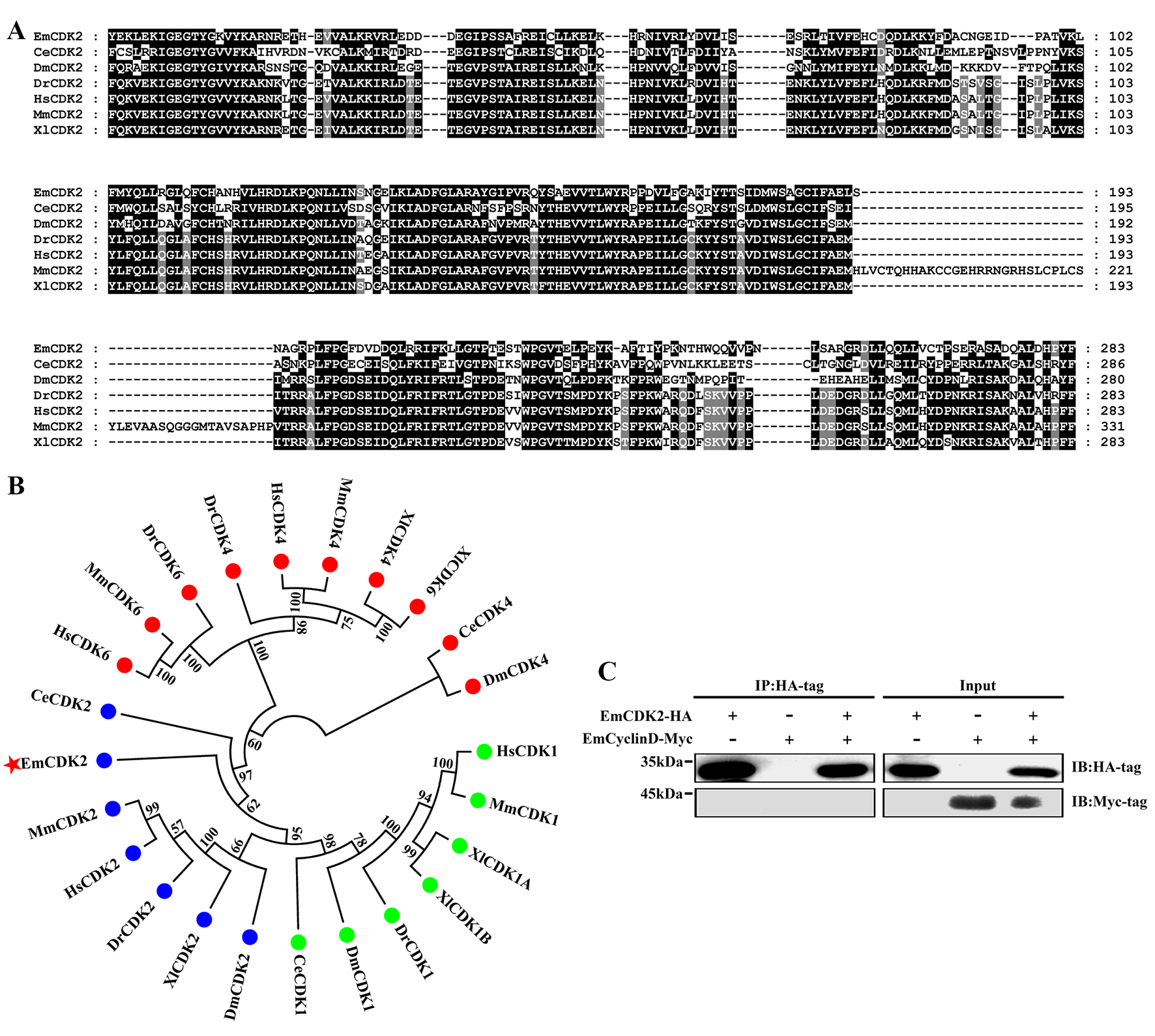

Supplement: Supplementary Figure 1 — Analysis of the CDK2 homolog of E. multilocularis amino acid sequence and the interaction with EmCyclinD. (A) Alignment of the S_Tkc domain of EmCDK2 with multiple species CDK2. (B) Phylogenetic analysis of EmCDK2 (marked with red star). The BLAST analyses of the S_Tkc domain of EmCDK2 were used for phylogenetic tree construction. (C) The Co-Immunoprecipitation of EmCyclinD and EmCDK2. Using an Anti-HA tag antibody for Immunoprecipitation. [file Image_1.TIF]

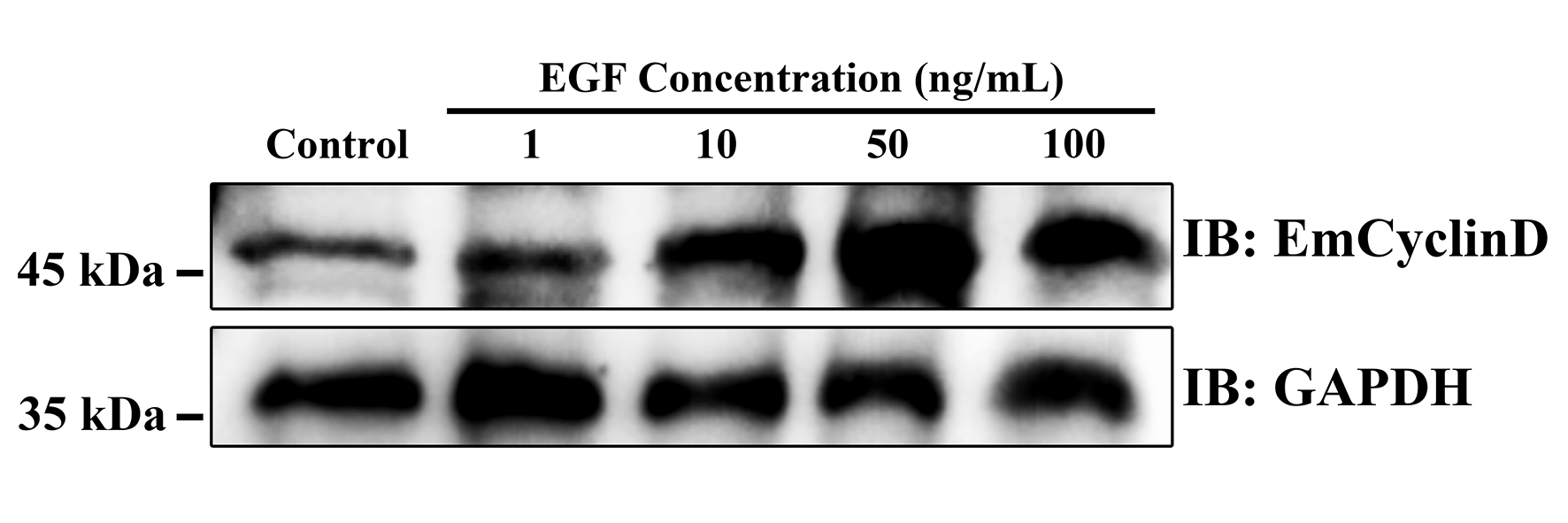

Supplement: Supplementary Figure 2 — Effects of EGF on EmCyclinD expression. Metacestode vesicles were cultured in DMEM without serum for 5 days, and then stimulated with 0 (Control) to 100 ng/mL recombinant human EGF for 45 min. [file Image_2.TIF]

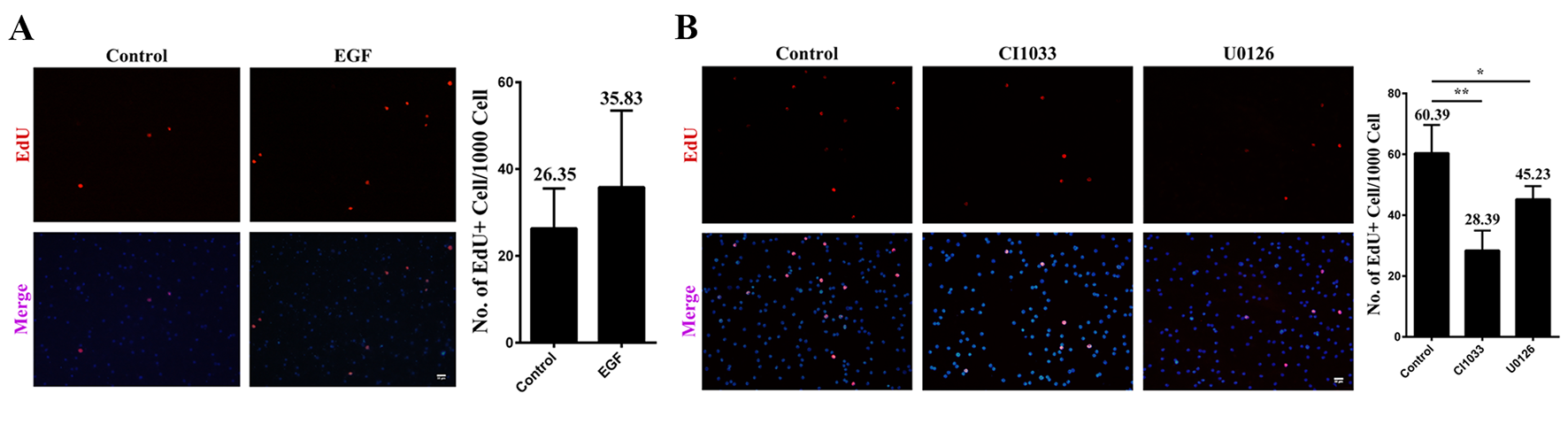

Supplement: Supplementary Figure 3 — The inhibition of EGFR-ERK inhibits the proliferation of germinative cells. (A) Host EGF stimulates the proliferation of germinative cells. Metacestode vesicles were pretreated with 40 mM of hydroxyurea for 3 days and allowed for a 4 days’ recovery in conditioned medium (control) supplemented with EGF. (B) CI-1033 and U0126 treatment inhibit germinative cell proliferation. Metacestode vesicles were treated with 10 μM CI-1033 or 40 μM U0126 for 4 days, Representative images are shown in the left panel, Bar = 50 μm. Quantification of EdU + cells is shown in the right panel. *P < 0.05, **P < 0.01. Data are shown as mean ± SD of at least three vesicles. [file Image_3.TIF]

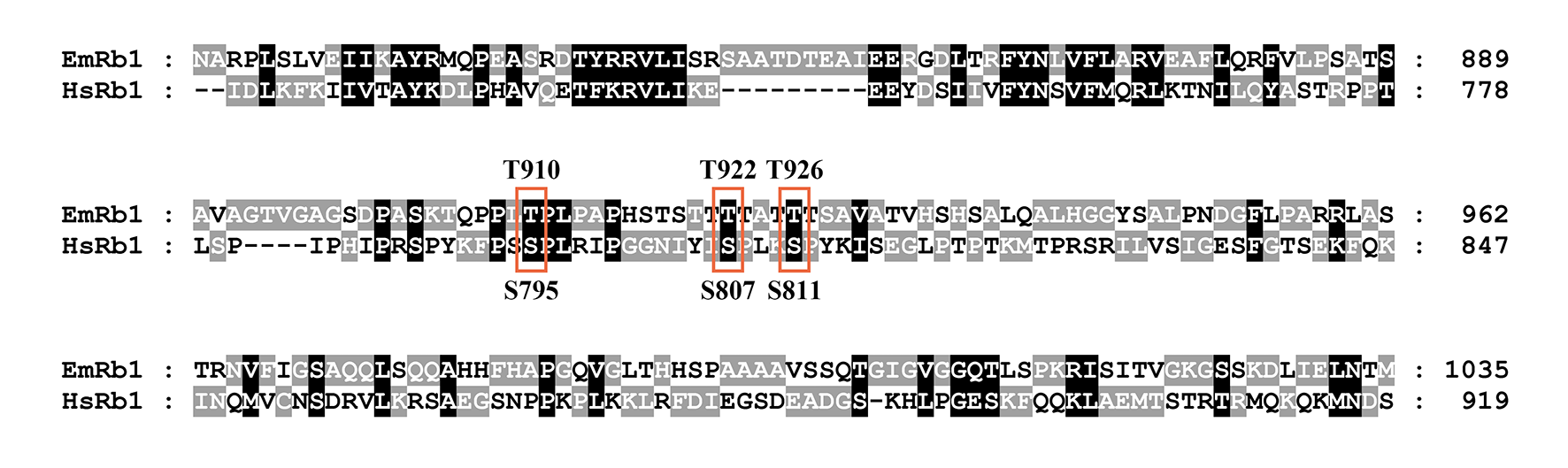

Supplement: Supplementary Figure 4 — Analysis of amino acid sequence of EmRb1. Alignment of the full length of EmRb1 with Human Rb1. Three CyclinD-CDK4/6 complex-related phosphorylation sites are conserved (Orange box). [file Image_4.TIF]
